# Suppression of the HBP Function Increases Pancreatic Cancer Cell Sensitivity to a Pan-RAS Inhibitor

**DOI:** 10.3390/cells10020431

**Published:** 2021-02-18

**Authors:** Francesca Ricciardiello, Laura Bergamaschi, Humberto De Vitto, Yang Gang, Taiping Zhang, Roberta Palorini, Ferdinando Chiaradonna

**Affiliations:** 1 Department of Biotechnology and Biosciences, University of Milano-Bicocca, 20126 Milan, Italy; francesca.ricciardiello@unimib.it (F.R.); laura.bergamaschi@istitutotumori.mi.it (L.B.); hdevitto@umn.edu (H.D.V.); 2 Department of General Surgery, Peking Union Medical College Hospital, Chinese Academy of Medical Sciences and Peking Union Medical College, Beijing 100730, China; 2010302180268@whu.edu.cn (Y.G.); andrewzhangt@aliyun.com (T.Z.)

**Keywords:** PDAC, hexosamine biosynthetic pathway, glycosylations, KRAS, KRAS inhibitors, cancer treatment

## Abstract

Pancreatic ductal adenocarcinoma (PDAC) is a leading cause of cancer-related death and the search for a resolutive therapy is still a challenge. Since KRAS is commonly mutated in PDAC and is one of the main drivers of PDAC progression, its inhibition should be a key strategy for treatment, especially considering the recent development of specific KRAS inhibitors. Nevertheless, the effects of KRAS inhibition can be increased through the co-inhibition of other nodes important for cancer development. One of them could be the hexosamine biosynthetic pathway (HBP), whose enhancement is considered fundamental for PDAC. Here, we demonstrate that PDAC cells expressing oncogenic KRAS, owing to an increase in the HBP flux, become strongly reliant on HBP for both proliferation and survival. In particular, upon treatment with two different compounds, 2-deoxyglucose and FR054, inhibiting both HBP and protein *N*-glycosylation, these cells undergo apoptosis significantly more than PDAC cells expressing wild-type KRAS. Importantly, we also show that the combined treatment between FR054 and the pan-RAS inhibitor BI-2852 has an additive negative effect on cell proliferation and survival by means of the suppression of both Akt activity and cyclin D1 expression. Thus, co-inhibition of HBP and oncogenic RAS may represent a novel therapy for PDAC patients.

## 1. Introduction

Pancreatic ductal adenocarcinoma (PDAC) is a leading cause of cancer-related death and is considered one of the most malignant and lethal cancers worldwide, with a five-year survival rate of less than 8% [1]. In addition, its incidence is rising [2] and it is projected to become the second leading cause of cancer-related death by 2030 [3]. Oncogenic mutations in KRAS are very frequent in PDAC, with the prevalence being around 90%, and this gene is usually already mutated in the early stages of cancer development [4]. Oncogenic KRAS plays a crucial role in tumor development and progression, promoting cell proliferation, cell death resistance, evasion from immune system, and metastasis; moreover, it contributes to the tumor microenvironment modifications and the typical cancer cell metabolic reprogramming [5,6]. Regarding the last process, several studies achieved in different cancer cell models have shown that oncogenic KRAS promotes glucose uptake and its use through glycolysis, favoring the Warburg effect [7]. Together with this, the KRAS-driven metabolic reprogramming also enables the enhancement of the glycolytic biosynthetic branches, such as the pentose phosphate pathway and the hexosamine biosynthetic pathway (HBP) [6,8].

HBP plays a crucial role in the cells, since it leads to the production of UDP-*N*-acetylglucosamine (UDP-Glc*N*Ac), the essential substrate for protein *N*- and *O*-glycosylation, a post-translational modification involved in protein folding, stability, localization, activity, and protein–protein interactions [9]. Therefore, nowadays, its fundamental role in many cellular processes is widely accepted, including those involved in tumor onset and development [10,11]. Accordingly, HBP flux enhancement and protein glycosylation increase and alteration have been found in different types of cancer [9], often as direct-driven mechanisms in cell proliferation, survival, epithelial–mesenchymal transition, metastasis, and chemotherapy resistance [12]. Indeed, different studies have demonstrated that the inhibition of the HBP and/or protein glycosylation induces cancer cell growth arrest and death [12]. In this regard, we recently synthesized and preclinically validated a new inhibitor called FR054, which targets phosphoglucomutase 3 (PGM3), the enzyme catalyzing the conversion of *N*-acetylglucosamine-6-phosphate (Glc*N*Ac-6P) in *N*-acetylglucosamine-1-phosphate (Glc*N*Ac-1P), and attenuates the HBP flux [13,14]. Importantly, FR054 is able to induce proliferation arrest and death in breast and pancreatic cancer cells and significantly reduces the growth of breast and pancreatic tumors in xenograft and PDX mice [14,15].

The direct targeting of the oncogenic KRAS protein is still in its infancy, but the future looks promising, particularly in light of recent progress [16]. Indeed, some specific compounds drugging wild type and oncogenic RAS or selectively drugging only specific mutant KRAS have been discovered and entered in clinical trials testing their efficacy on KRAS direct inhibition [17,18,19,20]. Nevertheless, these specific approaches also cause drug resistance, making necessary the identification of other strategies to avoid cancer cell adaptation [21,22,23]. Therefore, combined treatments able either to enhance the effects of the RAS inhibitors or to avoid the mechanisms of resistance are still needed [21,24]. Indeed, new combined treatments, targeting cell cycle-related pathways, DNA repair related pathways, and metabolic pathways have already been proposed and are currently being tested in preclinical and clinical trials [21].

Here we show that the oncogenic mutation of KRAS in PDAC cells, increasing their reliance on the HBP, also enhances their sensitivity to the inhibition of HBP and protein glycosylation. Furthermore, we show that this dependence may be used to significantly enhance the effect of a specific pan-RAS inhibitor on cell proliferation and death, suggesting a potential new therapeutic approach in PDAC. 

## 2. Materials and Methods

### 2.1. Cell Lines

PDAC cancer cell lines MIA PaCa-2 and PANC-1 were routinely cultured in high glucose Dulbecco’s modified Eagle’s medium (DMEM), while PDAC BxPC-3, SU.86.86, and Capan-1 were cultured in RPMI. In both cases, the medium was supplemented with 2 mM L-glutamine, 100 U/mL penicillin, 100 mg/mL streptomycin and 10–20% fetal bovine serum. Normal, transformed and reverted mouse fibroblasts NIH3T3 were routinely cultured in high glucose DMEM, supplemented with 4 mM L-glutamine, 100 U/mL penicillin, 100 mg/mL streptomycin and 10% newborn calf serum. The cells were grown and maintained according to standard cell culture protocols and kept at 37°C with 5% CO_2_. The medium was replaced every 2–3 days and cells were split or seeded for experiments when they reached the sub-confluence. 

### 2.2. Cell Treatments

Oxamate, 2-deoxyglucose (2-DG), and mannose were purchased from Sigma-Aldrich (Merck Life Science, Milan, Italy). FR054 was synthesized either by our laboratories or by WuXi AppTec Co., Ltd. (Tianjin, China) [13,14]. The RAS inhibitor BI-2852 was requested and obtained from Boehringer Ingelheim (Ingelheim am Rhein, Germany) [20].

### 2.3. Cell Viability and Cell Death Assays

To measure cell proliferation, harvested cells were counted using the Burker chamber. Where indicated, cell viable count was performed by staining cells with Trypan Blue 0.4% (Life Technologies - Thermo Fisher Scientific, Waltham, MA, USA). Cell viability was measured also with the MTT test (Roche) according to the manufacturer’s protocol.

Propidium iodide (PI)/annexin V-FITC staining was performed using the Apoptosis Assay Kit from Immunological Sciences and analyzed by a flow cytometer (CytoFLEX, Beckman Coulter Life Sciences, Indianapolis, IN, USA). Both the acquisition and the analysis of the data were performed with the CytExpert Software (version 2.4).

### 2.4. Western Blot

Cells were harvested and disrupted in appropriate lysis buffer; 10–50 μg of total proteins were resolved by SDS-PAGE and transferred to the nitrocellulose membrane, which was incubated overnight with specific antibodies: vinculin (sc-5573, 1:10,000), EIF2α (sc-133127, 1:1000), OGT (sc-32921, 1:200) and cyclin D1 (sc-20044, 1:1000) from Santa Cruz Biotechnology Inc. (DBA Italia, Segrate—Milan, Italy); cleaved caspase 3 (#9662, 1:500), CHOP (#2895, 1:1000), Grp78 (BiP, #3177, 1:1000), phospho-EIF2α Ser 51 (#3398, 1:1000), phospho-EGFR Tyr 1068 (#3777, 1:1000) and EGFR (#4267, 1:1000) from Cell Signaling Technology Inc. (Euroclone, Pero—Milan, Italy); α-*O*-Glc*N*Ac (Clone RL2, MA1-072, 1:1000) from Thermo Fisher Scientific; β-actin (A5441, 1:5000) and UAP1 (HPA014659, 1:250) from Sigma-Aldrich; GFPT1 (14132-1-AP, 1:1000) and PGM3 for human cells (A304-555A, 1:5000) from Bethyl Laboratories (Montgomery, TX, USA); phospho-Akt Ser 473 (MAB-94111, 1:1000) and Akt (MAB-10947, 1:1000) from Immunological Sciences (Società Italiana Chimici, Rome, Italy); and PGM3 for mouse cells (Ab128094, 1:200) from Abcam (Cambridge, UK). The protein signals following the enhanced chemiluminescent (ECL) reaction (Cyanagen, Bologna, Italy) were either detected on films (Amersham, GE Healthcare, Chicago, IL, USA) or captured by an imaging system (ChemiDoc XRS+ BIO-RAD, Hercules, CA, USA). Protein expression levels were quantified by densitometry analysis using the freely available ImageJ software.

### 2.5. Seahorse Analysis

The extracellular acidification rate (ECAR) throughout a glycotest assay and the oxygen consumption rate (OCR) throughout a mitostress test were determined using the Seahorse XF24 Analyzer (Agilent Technologies, Santa Clara, CA, USA). Briefly, for the analyses, the cells were seeded in a dedicated 24-well XF24 cell culture plate in a complete medium. Then, one hour before the analysis, this culture medium was exchanged for specific media for use throughout the experiment (unbuffered DMEM, supplemented with 2 mM glutamine for the glycotest assay or with 2 mM glutamine, 11 mM glucose, and 1 mM sodium pyruvate for the mitostress test). Different compounds were added in succession: glucose (10 mM), oligomycin (0.5 μM), and 2-DG (50 mM) for the glycotest assay; oligomycin (1 μM), FCCP (0.125 μM), and rotenone plus antimycin A (0.5 μM) for the mitostress test. The medium and compounds were purchased from Sigma-Aldrich. 

The XF Real-Time ATP Rate Assay was performed in the Seahorse XFe96 Analyzer (Agilent Technologies), following the XF protocol. The cells were seeded in 96-well XFe96 plates. After 24 h, for the analysis, this medium was exchanged for low buffered XF assay medium (103575-100, Agilent Technologies), supplemented with 2 mM glutamine, 10 mM glucose, and 1 mM sodium pyruvate. Oligomycin (0.5 μM) and rotenone plus antimycin A (1.5 μM) were added in succession and OCR, ECAR and proton efflux rate (PER) were measured. The data obtained were assessed with XF Wave Software (version 2.6.1), and this allowed the calculation of the ATP production rate due to the glycolysis (ATP Glyco) and the mitochondrial respiration (ATP Mito). 

After all analyses, the OCR and the acidification values obtained by the instrument were normalized according to the cell number. The energy map was built plotting the measurement of OCR and ECAR in basal condition.

### 2.6. Glucose and Lactate Measurement

D-glucose and L-lactate levels in culture medium were determined using spectrophotometric enzyme assay kits (Megazyme, Bray, Ireland) as specified by the manufacturer’s datasheet.

### 2.7. Membrane N-Glycosylation Analysis

To determinate the cell surface expression of *N*-linked glycoproteins, the cells were stained with 5 μg/mL Phaseolus vulgaris (PHA-L) Alexa Fluor 488 (FITC)-conjugated lectin (Thermo Fisher Scientific), diluted in a specific buffer (10 mM HEPES/NaOH pH 7.4, 140 mM NaCl, 2.5 mM CaCl_2_) and then analyzed by flow cytometry. Flow cytometric acquisitions and data analyses were performed using the CytoFLEX platform and the CytExpert software (Beckman Coulter). 

### 2.8. Confocal Microscopy Analysis of the Epidermal Growth Factor Receptor (EGFR) Localization at Endoplasmic Reticulum (ER) and Golgi Apparatus

The cells in different conditions were centrifuged on the coverslips (cytospin) and fixed with 4% paraformaldehyde for 6 min at room temperature. After that, the cell spots were permeabilized in 0.1% Triton X-100, blocked in 2% BSA, incubated with EGFR antibody (Cell Signaling #4267, 1:50), with secondary antibody (1:400), with Concanavalin-A (ConA) Alexa Fluor 594 (PE)-conjugated lectin (150 μg/μL) and then with dapi 2 μg/mL. Cells were mounted with dabco antifading reagent (Sigma-Aldrich) and then examined under an A1R Nikon laser scanning fluorescence confocal microscope (Nikon, Tokyo, Japan) at a magnification of 63X.

### 2.9. Statistical Analysis

Unless otherwise stated, the results are expressed as mean ± SEM of independent experiments carried out at least in triplicate. Differences between datasets were determined using Student’s *t*-test or one-way ANOVA test followed by either Tukey HSD (honestly significant difference) or Bonferroni post hoc tests. A *p*-value (*p*) < 0.05 was considered statistically significant.

## 3. Results

### 3.1. MIA PaCa-2 Cells Carrying Oncogenic KRAS Are More depEndent on Glycolysis, HBP and Glycosylation Processes Than KRAS Wild-Type BxPC-3

**Figure 1:**
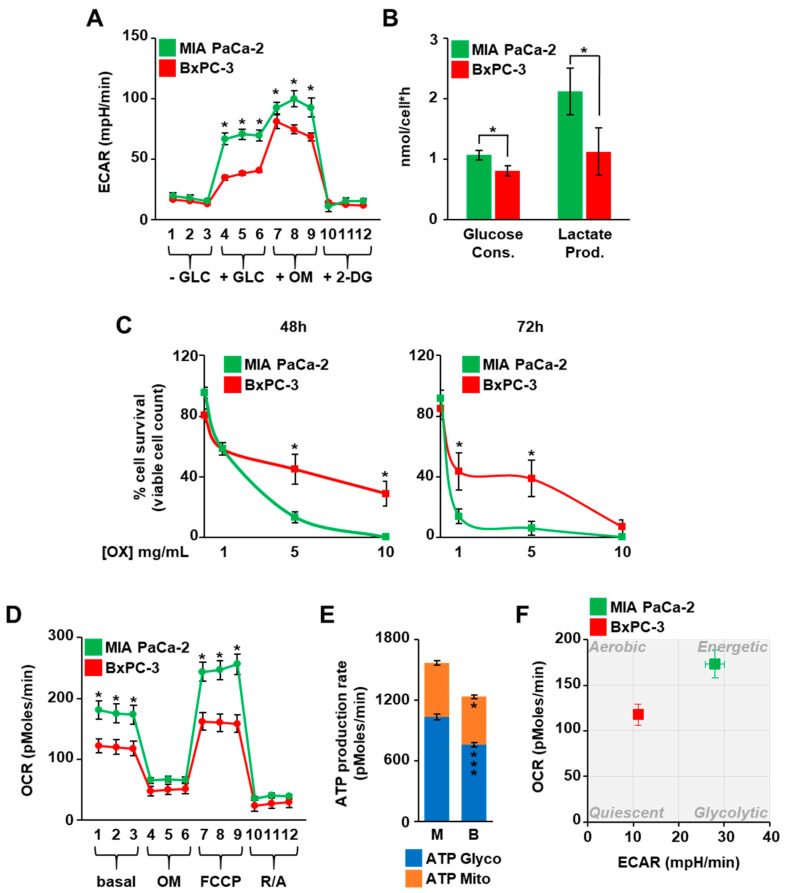
KRASmut MIA PaCa-2 cells are more glycolytic than KRASwt BxPC-3. (**A**) The extracellular acidification rate (ECAR) of MIA PaCa-2 and BxPC-3 cells throughout a glycotest assay was measured using the Seahorse XF24 Analyzer. (**B**) The glucose consumption and lactate production were calculated measuring the amount of glucose and lactate in cell media of MIA PaCa-2 and BxPC-3. (**C**) MIA PaCa-2 and BxPC-3 cells were cultured in presence of the glycolysis inhibitor oxamate (OX) and counted at the indicated time points using the Trypan Blue stain. (**D**) The oxygen consumption rate (OCR) of MIA PaCa-2 and BxPC-3 cells throughout a mitostress test was measured using Seahorse XF24 Analyzer. (**E**) The production rate of the ATP due to the glycolysis (ATP Glyco) and the mitochondrial respiration (ATP Mito) was evaluated in MIA PaCa-2 and BxPC-3 cells by performing the specific assay with the Seahorse XFe96 Analyzer. (**F**) The XF Energy Map was determined using the Seahorse profiles. All data represent the mean ± SEM of at least three independent experiments. * *p* < 0.05, *** *p* < 0.001 (Student’s *t*-test), MIA PaCa-2 vs. BxPC-3.

PDAC cells show different metabolic alterations, among which a relevant role has been assigned to glucose metabolism and its downstream anabolic pathways including the HBP [6,8]. Nevertheless, the specific role of the oncogenic KRAS as compared to wild type KRAS regarding the HBP changes observed in PDAC cells has been addressed less often. Therefore, to delineate such a role, we first compared the glycolytic flux and the rate of the glucose consumption and lactate production in BxPC-3 and MIA PaCa-2 cell lines, characterized by the expression of wild-type (KRASwt) and G12C-mutant KRAS (KRASmut), respectively. The ECAR, as measure of the glycolytic flux, was quantified by using the Seahorse technology. As shown in Figure 1A, both the basal glycolysis (+GLC) and the glycolytic capacity (+OM) were higher in MIA PaCa-2 cells than BxPC-3 cells. 

In addition, the glycolytic reserve (the difference between the glycolytic capacity and the basal glycolysis) was lower in MIA PaCa-2 cells, suggesting that the glycolysis was near to the maximum capacity in these cells. In accordance, MIA PaCa-2 cells presented a higher rate of both glucose consumption and lactate production than BxPC-3 cells (Figure 1B). A higher glucose consumption of MIA PaCa-2 cells was observed, while also measuring the residual glucose in the cell medium along a time course of 96 h (Figure S1A,B) and further confirmed by the faster glucose depletion from the medium of MIA PaCa-2 cells as compared with BxPC-3 cells (Figure S1C,D). To corroborate the major dependence of MIA PaCa-2 cells on glucose availability, both cell lines were treated with the lactate dehydrogenase (LDH) inhibitor oxamate, which is able to reduce the glycolytic flux [25]. As shown in Figure 1C, MIA PaCa-2 cells showed a significant greater sensitivity than BxPC-3, since a higher percentage of cell death in MIA PaCa-2 cells was observed. Similar results were obtained culturing cells upon glucose shortage. Indeed, comparing the low glucose condition (1 mM glucose as initial concentration) with the normal glucose condition (11 mM), MIA PaCa-2 cells showed earlier cell growth arrest as compared with BxPC-3 cells (Figure S1E,F). Altogether these observations confirmed the ability of the oncogenic KRAS to promote an increased glycolysis in PDAC cancer cells. Nevertheless, MIA PaCa-2 cells were characterized by a higher metabolic activity, since their oxygen consumption and ATP rate production, as measured by Seahorse technology, were also higher than in BxPC-3 cells (Figure 1D,E). Thus, comparing the ECAR and the OCR measured in normal conditions for both cell lines in an energy map, BxPC-3 cells showed an aerobic almost quiescent metabolism, while MIA PaCa-2 cells were characterized by a more glycolytic and energetic metabolism (Figure 1F). 

**Figure 2:**
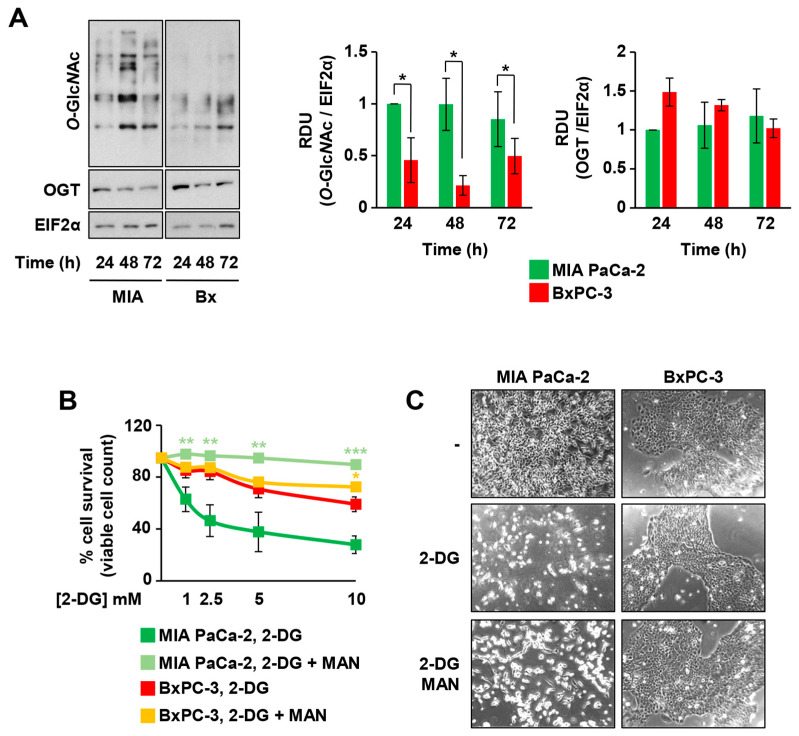
KRASmut MIA PaCa-2 cells are more dependent on the hexosamine biosynthetic pathway (HBP) and glycosylation for their survival than KRASwt BxPC-3. (**A**) Protein *O*-Glc*N*Ac and OGT expression were detected in total cell extracts from MIA PaCa-2 and BxPC-3 cells at 24, 48, and 72 h of culture. The expression of EIF2α was used for signal normalization. On the left, a representative image is shown, while the band intensity quantification of at least three independent blots is displayed in the histograms on the right. (**B**) MIA PaCa-2 and BxPC-3 cells were cultured in presence of different concentrations of 2-DG −/+ 1 mM mannose (MAN) and counted after 72 h using the Trypan Blue stain. (**C**) Representative microscopy images of MIA PaCa-2 and BxPC-3 cells upon 72 h-treatment with 2.5 mM 2-DG −/+ 1 mM MAN. All data represent the mean ± SEM of at least three independent experiments. * *p* < 0.05, ** *p* < 0.01 and *** *p* < 0.001 (Student’s *t*-test), −MAN vs. +MAN in panel (**B**).

Considering the previous observations and also that the KRAS-driven glycolytic metabolism is usually accompanied by the increase in the flux through the glycolytic branches, among which the HBP, the total protein *O*-glycosylation levels (*O*-Glc*N*Ac) were analyzed in MIA PaCa-2 and BxPC-3, as readout of the HBP flux. As shown in Figure 2A, the higher glucose consumption in MIA PaCa-2 cells was also associated with an enhanced level of protein *O*-Glc*N*Ac as compared with BxPC-3, despite a similar level of expression of *O*-linked β-*N*-acetylglucosamine transferase (OGT), the only enzyme deputies to intracellular protein *O*-Glc*N*Ac (Figure 2A). 

In order to better detail the influence of the HBP flux on the cell proliferation and survival of the two cell lines characterized by a different KRAS status, we treated both cell lines with 2-DG, which is able to inhibit glycolysis, HBP, and *N*-glycosylation process [26,27,28]. Indeed, 2-DG, decreasing the glucose uptake and inhibiting the glycolytic enzymes phosphoglucoisomerase and hexokinase, limits the fructose-6-phosphate synthesis, an essential substrate of the HBP [26,27,28], and competes with mannose for the protein *N*-glycosylation [29]. MIA PaCa-2 cells were significantly more sensitive to 2-DG treatment as compared with BxPC-3 since they showed a significant stronger and dose-dependent decrease in cell survival (Figure 2B) and cell proliferation (data not shown). Strikingly, the addition of 1 mM mannose, as a substrate to specifically enhance protein *N*-glycosylation, almost completely protected MIA PaCa-2 cells from 2-DG-dependent cell death (Figure 2B,C). Altogether, these findings suggest that MIA PaCa-2 cells, being more dependent on glycolytic flux and HBP, are significantly protected by mannose, hence confirming the relevant role of protein *N*-glycosylation in KRASmut cells. Notably, the detrimental effect of 2-DG in MIA PaCa-2 cells was also preserved by the addition of 10 mM Glc*N*Ac, a specific substrate able to enhance the HBP flux, further confirming their great dependence on the HBP (Figure S2A). Importantly, the measurement of the released lactate in the medium, as an indication of the glycolytic flux, in MIA PaCa-2 cells treated with 2-DG and/or mannose indicated that only 2-DG, as expected, impacted the lactate production, leading to a significant decrease. Mannose did not influence the rate of the lactate release in both untreated and 2-DG-treated cells (Figure S2B), confirming that the pro-survival effect of mannose is not dependent on glycolysis refueling. To further expand previous findings, we evaluated the metabolic characteristics and the effects of 2-DG- and mannose-treatments in other PDAC cancer cell lines characterized by expression of oncogenic KRAS, namely PANC-1 (G12D), SU.86.86 (G12D), and Capan-1 (G12V), and derived either from primary tumor (PANC-1) or from metastasis (SU86.86 and Capan-1). To characterize their metabolism, we first measured their ATP rate production and the ECAR throughout a glycotest assay. As shown in Figure S3A,B, the three cell lines showed different profiles of ATP rate production and ECAR. In particular, PANC-1 cells were similar to MIA PaCa-2 cells, since they had a higher energetic metabolism (Figure S3A) and glycolytic activity (Figure S3B) as compared to the other two cell lines. Differently, SU.86.86 appeared more glycolytic and Capan-1 showed a mixed energetic metabolism, being equally glycolytic and respirative and, in general, less metabolically active. Since published data indicate that different KRAS mutations may give rise to different changes in cancer metabolism and phenotype [30,31], we decided to exactly address in these cell lines the *O*-Glc*N*Ac level, the HBP’s enzyme expression, and the sensitivity to 2-DG and mannose treatment. As shown in Figure S4A, the more glycolytic PDAC cell lines, MIA PaCa-2 and PANC-1, had higher levels of protein *O*-Glc*N*Ac than BxPC3, SU.86.86, and Capan-1. Nevertheless, except for OGT, the KRASmut cell lines had higher levels of different HBP enzymes, i.e., GFPT1, PGM3, and UAP1 (Figure S4B), suggesting an enhancement of the HBP pathway and protein glycosylation. Indeed, as previously observed in MIA PaCa-2 cells, all KRASmut PDAC cell lines were sensitive to 2-DG treatment in a dose-dependent manner, since both cell number and death were significantly affected (Figure S4C,D). Importantly, albeit with a different value of fold change, mannose treatment rescued their proliferation and survival, further confirming that oncogenic KRAS expression enhances HBP dependence in PDAC cell lines. 

### 3.2. 2-DG Treatment Induces a Decrease in N-Glycosylation and Activation of the Unfolded Protein Response That Is Restored by Mannose Treatment Specifically in KRASmut Cells

**Figure 3:**
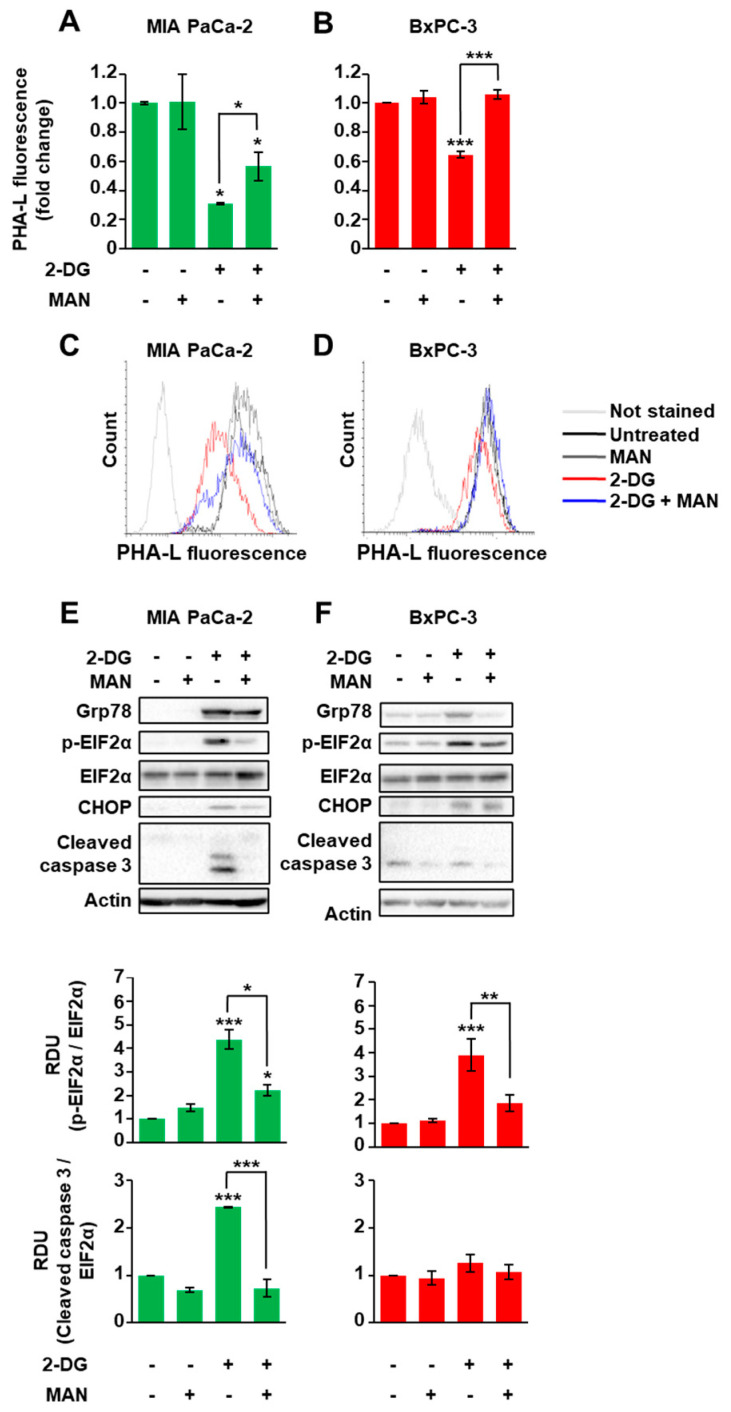
KRASmut MIA PaCa-2 cells are more sensitive than KRASwt BxPC-3 to the alteration of the *N*-glycosylations and the resulting endoplasmic reticulum (ER) stress. (**A**–**D**) Protein *N*-glycosylations were detected in viable MIA PaCa-2 (**A**,**B**) and BxPC-3 (**C**,**D**) cells after 72 h treatment with 2.5 mM 2-DG −/+ 1 mM mannose (MAN). In particular, tri-/tetra-branched *N*-glycans were recognized by FITC-conjugated PHA-L. The quantification of the mean fluorescence of independent experiments is displayed in the histograms (**A**,**B**), while representative flow cytometric profiles are shown in panels (**C**,**D**). (**E**,**F**) The expression of the UPR markers was analyzed through Western blot in MIA PaCa-2 (**E**) and BxPC-3 (**F**) cells treated with 2.5 mM 2-DG −/+ 1 mM MAN. Actin was used for signal normalization. The images are representative of at least three independent blots. The quantification of the EIF2α phosphorylation and Cleaved Caspase 3, considering all experiments performed, is displayed in the histograms on the bottom. All data represent the mean ± SEM of at least three independent experiments. * *p* < 0.05, ** *p* < 0.01 and *** *p* < 0.001 (One-way ANOVA), untreated vs. treated where not specifically indicated.

Previous data indicated that several membrane proteins, such as tyrosine kinase receptors, needs to be modified by complex branched *N*-glycans in order to support cancer cells’ proliferation and survival [32]. Therefore, we evaluated whether 2-DG- and mannose-treatments could impact on cell membrane protein *N*-glycosylation levels. We used a specific lectin, PHA-L, which is able to recognize the complex tri-/tetra-branched *N*-glycans structures, vastly dependent on the rate of the HBP flux. As shown in Figure 3A–D, upon 2-DG treatment, the PHA-L staining was significantly decreased in MIA PaCa-2 cells (around 70% of reduction) as compared to BxPC-3 cells (around 34% of reduction). Strikingly, such a reduction was significantly blunted by mannose addition in both cell lines; however, as previously shown in Figure 2B, only in MIA PaCa-2 cells was the mannose-dependent recovery of protein *N*-glycosylation associated with cell survival. 

Our previous results indicate that glucose depletion, affecting the HBP flux, causes a reduction in protein glycosylation that leads first to an ER stress response, known as unfolded protein response (UPR), and then to cell death [33]. Therefore, we sought to determine whether 2-DG and mannose treatments could modulate UPR and cell death depending on the cell KRAS mutagenic status. For this purpose, MIA PaCa-2 and BxPC-3 cells were treated for 72 h with 2.5 mM 2-DG alone or in combination with mannose and then analyzed for the expression levels of some UPR markers, including glucose-regulated protein of 78 kDa (Grp78), C/EBP homologous protein (CHOP), and the phosphorylation of eukaryotic translation initiation factor 2A (p-EIF2α), as well as for caspase 3 activation as a readout of cell death. 2-DG treatment enhanced Grp78 and CHOP protein levels as well as EIF2α phosphorylation in both cell lines (Figure 3E,F), validating the UPR activation. Conversely, the caspase 3 activation was significantly enhanced only in MIA PaCa-2 cells (Figure 3E,F). Remarkably, upon mannose co-addition, both cell lines showed the attenuation of the UPR marker expression, but only in MIA PaCa-2 cells was a complete inhibition of caspase 3 activation observed. Altogether, these findings indicate the greater sensitivity of the KRASmut PDAC cells to the UPR activation that follows the alteration of the *N*-glycosylations, as compared to BxPC-3 cells, which instead appear more able to cope with this ER stress response. 

### 3.3. KRASmut MIA PaCa-2 Cells Are Dependent on the HBP 

**Figure 4:**
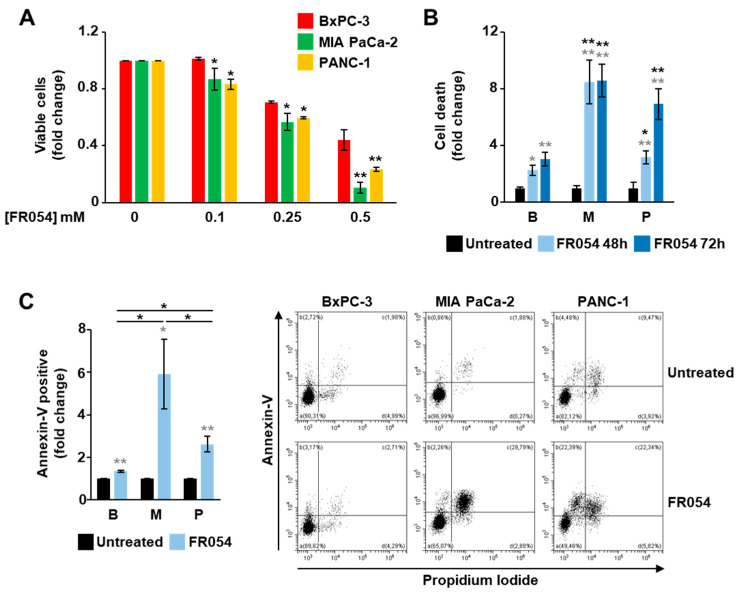
KRASmut MIA PaCa-2 and PANC-1 cells are more sensitive to the HBP inhibition mediated by FR054 than KRASwt BxPC-3. (**A**) Cell viability of BxPC-3, MIA PaCa-2 and PANC-1 cells treated for 48 h with different concentrations of FR054 was detected by viable count using Trypan Blue stain. (**B**,**C**) Cell death of BxPC-3, MIA PaCa-2 and PANC-1 treated with 0.5 mM FR054 was evaluated by viable count (**B**) and by using flow cytometric analysis of Annexin V-FITC and propidium iodide staining (**C**). Representative profiles of the flow cytometric analysis are shown on the right of panel (**C**). All data represent the mean ± SEM of at least three independent experiments. * *p* < 0.05 and ** *p* < 0.01 (Student’s *t*-test in panel (**A**); one-way ANOVA in panels (**B**,**C**)), MIA PaCa-2 and Panc-1 vs. BxPC-3 (black asterisks) and untreated vs. treated (grey asterisks) where not specifically indicated.

To further detail the role of the KRAS status in the dependence of the PDAC cells on HBP and *N*-glycosylation processes, we took advantage of an HBP inhibitor previously generated by our group, named FR054 [13], which is able to inhibit the HBP enzyme PGM3 causing a reduction in the HBP flux and consequently a significant attenuation of both protein *N*- and *O*-glycosylations [14,15]. MIA PaCa-2, PANC-1 and BxPC-3 cell lines were treated for 48 h with different concentrations of FR054 and then the cell viability and death were quantified. Both cell proliferation (Figure 4A) and cell death analysis (Figure 4B) indicated that MIA PaCa-2 and PANC-1 cells were more sensitive to HBP inhibition as compared with BxPC-3.

These findings were further corroborated by the cytofluorimetric analysis of the cell death upon 48 h treatment with 0.5 mM FR054 (Figure 4C). Indeed, the annexin V-FITC staining indicated that FR054 treatment in MIA PaCa-2 and PANC-1 cells caused a significant increase in apoptotic cells (Annexin V-positive) as compared to untreated cells and BxPC-3 cells. To further confirm the association between cell death and the *N*-glycosylation reduction following the treatment with FR054, the most sensitive MIA PaCa-2 cells were co-treated with FR054 and mannose and evaluated in terms of cell death. As shown in Figure S5, the treatment with mannose significantly protected the cells from the detrimental effect of FR054, confirming the tight connection between HBP flux, *N*-glycosylation, and cell death in KRASmut PDAC cells. 

### 3.4. The Combined Treatment with Drugs Inhibiting RAS and HBP Is Detrimental for Cells Expressing Oncogenic KRAS

**Figure 5:**
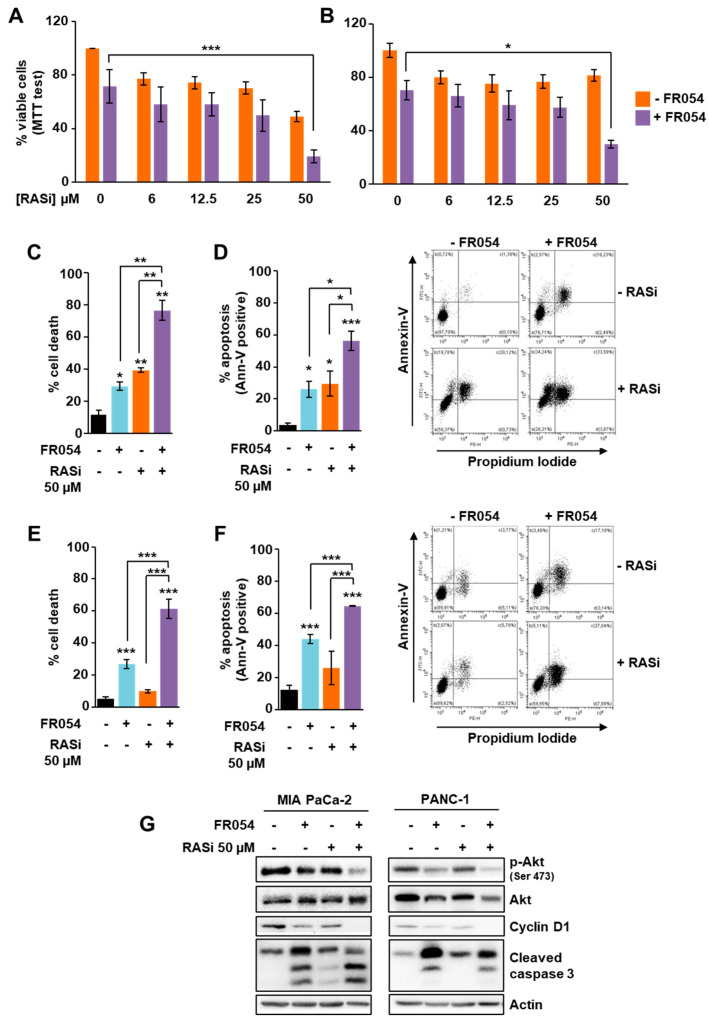
In KRASmut MIA PaCa-2 and PANC-1 cells, FR054 and the pan-RAS inhibitor BI-2852 present additive negative effects on cell proliferation and survival. (**A**,**B**) Cell viability of MIA-PaCa-2 and PANC-1, treated for 72 h with FR054 (350 µM for MIA-PaCa-2, 500 µM for PANC-1) and different doses of RAS inhibitor BI-2852 (RASi), was detected through MTT test. (**C**–**G**) MIA PaCa-2 and PANC-1 were treated with FR054 and 50 µM BI-2852 for 72 h. (**C**–**E**) Cell death was evaluated by viable count using Trypan Blue stain (**C**,**E**) and by using flow cytometric analysis of annexin V-FITC and propidium iodide staining (**D**,**F**). Representative profiles of the flow cytometric analysis are shown on the right of the panels (**D**,**F**). (**G**) The protein expression was analyzed through Western blotting. Actin was used for signal normalization. All data represent the mean ± SEM of at least three independent experiments. * *p* < 0.05, ** *p* < 0.01 and *** *p* < 0.001 (One-way ANOVA), untreated vs. treated where not specifically indicated.

Some specific inhibitors of the mutant KRAS have been developed in recent years, and they are considered promising for cancer treatment. Nevertheless, such targeting of mutant KRAS does not seem totally efficient yet, requiring the parallel inhibition of other targets as a resolved strategy [16,34]. Considering the strong reliance of KRASmut cells on the HBP and glycosylation, we decided to evaluate the effect of the combined treatment between FR054 and the pan-RAS inhibitor BI-2852 [20] in KRASmut cells MIA PaCa-2 and PANC-1. First, we evaluated the effect on cell proliferation and viability of different micromolar doses of BI-2852 alone or combined with FR054. As shown in Figure 5A and Figure S6A, in MIA PaCa-2 cells BI-2852 inhibited the cell proliferation in a dose-dependent manner until reaching almost 50% inhibition at the highest dose (50 µM). In contrast, in PANC-1 cells (Figure 5B), BI-2852 had a little effect at all doses (around 20% reduction at 50 µM), confirming previous data indicating this cell line as significantly resistant to genetic or pharmacological inhibition of KRAS [35,36]. 

To further detail the effect of the single and combined treatments, we measured cell death by Trypan Blue assay and propidium iodide/annexin V-FITC staining in both MIA PaCa-2 and PANC-1 cells. In MIA PaCa-2 cells both inhibitors, as previously observed in terms of proliferation, were able to increase cell death either alone or in combination (Figure 5C,D and Figure S6B,C). Conversely, in PANC-1 cells, only FR054 was able to induce cell death (Figure 5E,F). Indeed, as already observed in terms of proliferation, the effect of BI-2852 was slight, confirming PANC-1’s resistance. Importantly, the combined treatment strongly affected their cell viability, inducing a significant enhancement of the cell death as compared with untreated and single-treated samples (Figure 5E,F). Importantly, the analysis of cell proliferation and death in KRASwt BxPC3 cells, after single or combined treatment with FR054 and BI-2852 (Figure S7), indicated that this cell line was as sensitive to the RAS inhibitor as MIA PaCa-2 and as previously shown [37], but such an effect was not significantly enhanced by FR054 co-treatment, strengthening the observation of their lower dependence on HBP flux. In contrast, proliferation and viability analyses in SU.86.86 and Capan-1 confirmed the sensitivity of KRASmut cell lines to HBP inhibition by FR054 either as a single or combined treatment with the RAS inhibitor BI-2852 (Figure S8A,B), further confirming the role of the HBP flux in KRASmut PDAC cell lines in proliferation and survival. To investigate the molecular impact of the single and combined treatments on KRASmut cells, we analyzed the expression and the activity levels of some proteins linked to RAS signaling, as well as cleaved caspase 3 as apoptotic marker. As shown in Figure 5G, in MIA PaCa-2 cells and PANC-1 cells, the caspase 3 activation was significantly stronger in the combined treatment as compared to single ones, confirming the previous data on cell viability. Then, we analyzed two downstream effectors of RAS, cyclin D1, whose levels are primarily induced by the RAS- extracellular signal-regulated kinase (ERK) pathway, and Akt, whose phosphorylation and activation are induced by the RAS-phosphatidylinositol 3-kinase (PI3K) pathway. Both the expression of cyclin D1 and the activation of Akt, measured as phosphorylation at Ser 473, were diminished in combined treatment as compared to untreated and single drug-treated cells (Figure 5G), hence confirming that the combined treatment is able to arrest proliferation and pro-survival signaling. To further detail the effect of FR054 and BI-2852 on cell signaling, we also analyzed EGFR activation and localization since it is known that the inhibition of the EGFR glycosylation is detrimental for its activity and membrane localization [15,38,39,40]. Western blot analysis of the EGFR activation, evaluated through the phospho-EGFR Tyr 1068 level, in MIA PaCa-2 cells indicated that FR054 treatment induced a significant decrease in both untreated and BI-2852-treated cells (Figure S9A). In addition, the immunostaining analysis of the EGFR localization in permeabilized cells indicated that FR054 caused the accumulation of the receptor mainly in the ER and Golgi apparatus (detected by using fluorescent ConA, which is considered a marker of both compartments) as compared with untreated cells, where the EGFR was also present on the membrane (Figure S9B). Altogether, these findings suggest that HBP inhibition, by reducing the *N*-glycosylation, induces UPR activation, pro-survival signaling attenuation and cell death increase that concurrently significantly enhance the effectiveness of the pan-RAS inhibitor in KRASmut PDAC cells.

### 3.5. KRAS Mutation Is the Major Determinant for HBP Reliance of Cancer Cells

**Figure 6:**
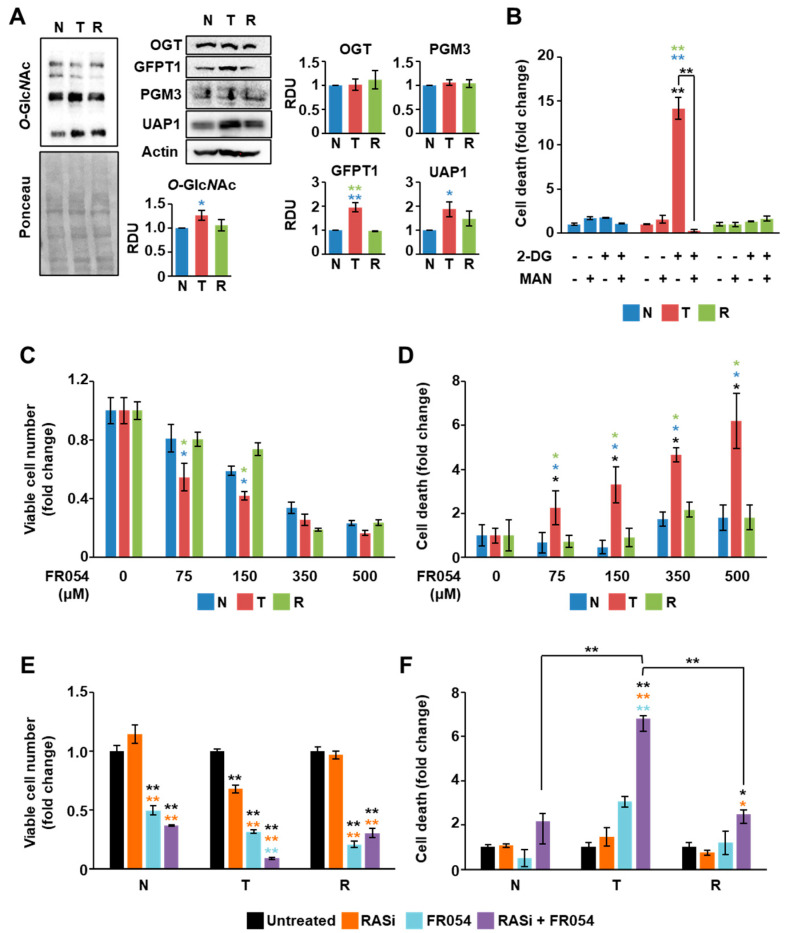
Oncogenic KRAS expression in NIH3T3 cells induces sensitivity to HBP and RAS inhibition. (**A**) Protein *O*-GlcNAc levels as well as the expression of the HBP proteins were analyzed through Western blotting in normal (N), transformed (T) and reverted (R) NIH3T3 cells. As a loading control, Ponceau staining and actin expression were used, respectively. Representative images are shown, while the band intensity quantification of at least two independent blots is displayed in the histograms. (**B**–**F**) Cell number (**C**,**E**) and cell death (**B**,**D**,**F**) of the NIH3T3 cells were evaluated through Trypan Blue assay upon 72 h treatment with 2.5 mM 2-DG −/+ 1 mM mannose (**B**), with different concentrations of FR054 (**C**,**D**), and with 350 µM FR054 −/+ 50 µM BI-2852 (**E**,**F**). Data represent the mean ± SEM of at least two independent experiments. * *p* < 0.05, ** *p* < 0.01 (one-way ANOVA), untreated vs. treated and T vs. N or R (see colored asterisks), where not specifically indicated.

To finally assess the contribution of KRAS mutation in the previously observed phenotypes, we took advantage of a well-established isogenic cell model of immortalized NIH3T3 mouse fibroblasts transformed by homologous recombination with G12V-mutant KRAS, referred to from now on as transformed (T) cells, as well as of an isogenic model of oncogenic KRAS attenuation, referred to from now on as reverted (R) cells, stably expressing a GEF dominant negative able to attenuate the oncogenic activation of the G12V-mutant KRAS cell line [41,42]. As a control, we used the NIH3T3 cells, referred to from now on as normal (N) cells, a genetically well-characterized immortalized cell line used as a model of “normal” cells to study transformation. Importantly, by using this isogenic cell model, we previously showed that transformed cells exhibit elevated sensitivity to glucose availability, reduced mitochondrial function, and reduced proliferation in response to glucose, glutamine, or galactose shortage as compared with normal and reverted cell lines [33,43,44,45,46,47,48,49]. Therefore, we performed analyses of different parameters, among which the protein *O*-glycosylation level, the HBP’s enzyme expression at basal level, and the sensitivity to 2-DG, mannose, FR054, and pan-RAS inhibitor. As shown in Figure 6A, transformed cells showed higher *O*-glycosylation level as well as GFPT1 and UAP1 expression as compared to normal and reverted cell lines, suggesting a higher HBP flux in this KRASmut cell line.

Importantly, they were also significantly more sensitive to 2-DG treatment as compared to the other cell lines, and the effect of the inhibitor was completely blunted by the co-treatment with mannose (Figure 6B). Furthermore, transformed cells were more sensitive to the HBP inhibitor FR054, especially in terms of cell death (Figure 6C,D), and, when treated with the combination of FR054 and the pan-RAS inhibitor BI-2852, they showed also a significant proliferation arrest and cell death (Figure 6E,F). Strikingly, almost all these effects appeared completely repressed in normal and reverted cell lines, indicating that the attenuation of the oncogenic KRAS-phenotype reverts the characteristic dependence on the HBP observed in transformed cells.

## 4. Discussion

The challenge of finding a therapy for pancreatic cancer is still open and, despite the increasing knowledge about this tumor and its molecular features, a tool for the treatment does not exist yet [34]. PDAC is characterized by a high frequency of mutations in KRAS; thus, the development of strategies drugging the oncogene may be promising for its treatment [50]. In recent years, breakthroughs have been made in the identification of new drugs targeting mutant RAS as well as approaches to blocking its cancer-related signaling. Nevertheless, a great amount of work remains to be done, and the investigation of different strategies is crucial. In the last few years, cancer remodulation of the cell metabolism has been increasingly studied by oncology research for exploitation for therapy [51]. Among the metabolic pathways remodulated in cancer, the increase in the HBP flux accompanied by the alteration of protein *N*- and *O*-glycosylations has been individuated as crucial [12,52]. Specifically, in PDAC, the HBP, the driver metabolic pathway for protein glycosylation, is enhanced by hypoxia and/or oncogenic KRAS activation, and such enhancement is linked to the hyperactivation of the glycolysis [8,53]. On the other hand, HBP stimulation has been also observed under moderate glucose and amino acid shortage, suggesting its adaptive role to enhance cell survival under harsh conditions, which are characteristic in PDAC [54]. Although it is well-established that the oncogene KRAS is the main driver for the acquisition of several cancer features by PDAC cells, whether this oncogene has a relevant role also in the HBP enhancement as compared with PDAC cells expressing wild type KRAS has been less well-addressed and still needs more clarifications. To elucidate this role, in our study we first took advantage of two PDAC cell lines characterized by the expression of wild type KRAS, BxPC-3 cells, and mutant KRAS, MIA PaCa-2 cells. Consistent with results from previous studies [55], we found that MIA PaCa-2 cells present a glycolytic phenotype and consequently are more sensitive than KRASwt BxPC-3 cells to the inhibition of the aerobic glycolysis achieved by using oxamate or 2-DG. However, previous data indicated that 2-DG can also affect other glucose-dependent metabolic pathways, including the HBP, and *N*-glycosylation [56]. Accordingly, here we show that, regardless of the KRAS status, 2-DG treatment mediates a decrease in the membrane-localized complex *N*-glycans (tri-/tetra-branched), whose levels are very sensitive to alteration in the HBP flux and UDP-Glc*N*Ac levels [57]. Importantly, the effect is more consistent in MIA PaCa-2 cells than in BxPC-3 cells and, in association with the stronger reduction in protein glycosylation, the KRASmut cells display a drastic enhancement of cell death not observed in KRASwt BxPC-3 cells. Importantly, such sensitivity to 2-DG was also found also other PDAC cell lines characterized by the expression of the oncogenic form of KRAS, namely PANC-1, SU.86.86, and Capan-1. Of note, although these three cell lines presented differences regarding their metabolic features, as previously published [55], all demonstrated a great vulnerability to 2-DG. Importantly, we also show that mannose, able to refuel protein *N*-glycosylation, can preserve KRASmut cells from the cell death induced by 2-DG, revealing that such oncogenic KRAS-dependent cell death is deeply associated with the reduction in protein *N*-glycosylation. Indeed, the inhibition of the HBP flux by means of the FR054, a compound able to inhibit HBP and *N*-glycosylations [14,15], equally resulted in stronger cell death in the KRASmut cells MIA PaCa-2 and PANC-1 than in KRASwt BxPC-3 cells. Importantly, FR054-dependent cell death was also inhibited in MIA PaCa-2 cells, by mannose treatment, further underlining the relevant role of *N*-glycosylation in KRASmut cell survival. Notably, all these findings were also confirmed in a well-established model of KRAS transformation. Indeed, we show that KRAS-transformed NIH3T3 fibroblasts, as compared to normal and reverted NIH3T3 cells, are more sensitive to 2-DG and to mannose protection as well as being more sensitive to FR054 treatment, confirming in this cell model the pro-survival role of the HBP in KRASmut cells.

The different sensitivity to the HBP inhibition between KRASmut and KRASwt PDAC cell lines could be due to several reasons, including differences in the membrane *N*-glycan profiles, as previously shown in other PDAC cells [58], since less complex *N*-glycan structures, linked to a less aggressive cancer phenotype, are also less sensitive to Glc*N*Ac level fluctuation [57,59]. In fact, the oncogenic RAS causes an increase in the beta1,6 branching of complex *N*-glycans structures (recognized by PHA-L), as previously shown by other authors [60,61], as well as the increase in the protein *O*-Glc*N*Acylation not associated to changes in the OGT expression, as we show, persuasively suggesting an enhancement of the HBP flux in KRASmut pancreatic cells. Indeed, published results indicate that oncogenic KRAS may induce higher levels of the HBP end products UDP-Glc*N*Ac plus UDP-Gal*N*Ac (UDP-Hex*N*Ac) [62], further confirming the close association between the oncogene expression and the HBP flux. The different sensitivity of the cell lines can also be justified by their different ability to cope with the downstream effects induced by HBP inhibition. Indeed, as demonstrated here and elsewhere [33,63], cancer cells carrying oncogenic KRAS are less able to cope with the ER stress caused by the disruption of the *N*-glycosyations as compared with KRASwt cells, which undergo UPR-mediated apoptosis. Therefore, based on previous reports and the data shown here, we propose a model in which oncogenic KRAS induces a significant reliance on HBP flux, which may be exploited for the PDAC therapy.

Recent data indicated that, while a certain variability exists in terms of the anti-tumor responses to genetic ablation or pharmacological inhibition of KRAS [22,64,65,66], the targeting of KRAS still remains a great therapeutic opportunity, especially in the context of combination therapies that may circumvent cell-specific resistance. Considering the high dependence of PDAC KRASmut cells on the HBP, the use of drugs able to modulate this metabolic pathway could be a good strategy for therapy, particularly in combination with compounds inhibiting RAS. As widely described, adaptation to endogenous KRAS ablation may activate several pathways in PDAC cells including epithelial-mesenchymal transition, integrin, and receptor tyrosine-kinase (RTK) signaling as well as cell cycle-related pathways such as cyclin-dependent kinase 4 (CDK4)/cyclin D1 [22,24,64,65,66]. In this regard, our previous data indicated that HBP inhibition, achieved through cancer cell treatment with the FR054, causes the decrease in the integrin membrane localization, cell adhesion, and migration, as well as the inhibition of the EGFR and Akt signaling [15]. Thus, since several of these pathways, alone or in combination, represent possible mechanisms of resistance to KRAS ablation, we believe that FR054 could enhance the activity of RAS inhibitors in PDAC cells. Indeed, here we show that the combined treatment with the HBP inhibitor FR054 and the pan-RAS inhibitor BI-2852 causes an additive detrimental effect in KRASmut PDAC cells that is accompanied by a substantial decrease in the RAS-downstream signaling. In particular, we show that the inhibitory effect of BI-2852, especially on cyclin D1 expression, is further enhanced by FR054. This result is highly interesting in the light of the fact that beyond its role as a marker of cancer proliferation and progression [67], cyclin D1 has been associated to cancer cell resistance to KRAS inhibitors [68]. Accordingly, it has been shown in PDAC cells and in other cancer types, that the co-inhibition of the cyclin D1 interacting kinases, CDK4/6, potentiates the global effects of KRASG12C inhibition, blocking the cell proliferation and inducing tumor regression [68,69]. Strikingly, such a reduction in cyclin D1 is also linked to a specific effect of FR054, which in single or combined treatment is able to affect pro-survival and proliferative mechanisms such as EGFR and Akt activation. Importantly, EGFR signaling activation has been recently shown to be an important escape mechanism upon KRAS inhibition or gemcitabine treatment in lung cancer and PDAC cancer, respectively [15,22,70]. Furthermore, it has been also shown that the inhibition of protein *N*-glycosylation may disrupt the RTK-driven signaling and sensitize cancer cells to chemotherapeutic drugs [15,32,70].

Therefore, since the evaluation of useful mechanisms to counteract cellular escape from RAS inhibition is a strong field of interest, we believe that our findings regarding the dependence of KRASmut cells on HBP and glycosylation processes should be considered a promising strategy to make targeting oncogenic RAS in PDAC cells more effective.

## Supplementary Materials

The following are available online at https://www.mdpi.com/2073-4409/10/2/431/s1, Figure S1: KRASmut MIA PaCa-2 cells show a higher glucose consumption than KRASwt BxPC-3; Figure S2: Glc*N*Ac and mannose protect MIA PaCa-2 cells upon 2-DG treatment, without affecting the glycolysis; Figure S3: Metabolic profile of PDAC cells; Figure S4: Mannose protects KRASmut cells upon 2-DG treatment; Figure S5: Mannose protects MIA PaCa-2 cells upon FR054 treatment; Figure S6: Combined 48h-treatment of KRASmut MIA PaCa-2 cells with FR054 and the pan-RAS inhibitor BI-2852; Figure S7: Combined treatment of KRASwt BxPC-3 cells with FR054 and the pan-RAS inhibitor BI-2852; Figure S8: Combined treatment of KRASmut SU.86.86 and Capan-1 cells with FR054 and the pan-RAS inhibitor BI-2852; Figure S9: Upon treatment with FR054 EGFR accumulates at ER and Golgi apparatus.
