# Supplementary material for: Suppression of the HBP Function Increases Pancreatic Cancer Cell Sensitivity to a Pan-RAS Inhibitor
Source: Cells. 2021 Feb 18;10(2):431. doi: 10.3390/cells10020431 (PMC7923121; doi:10.3390/cells10020431)
Supplement: Supplementary file 1 [file cells-10-00431-s001.pdf]

Suppression of the HBP function increases pancreatic cancer cell sensitivity to a pan-RAS inhibitor

Francesca Ricciardiello[1], Laura Bergamaschi[1,2], Humberto De Vitto[1,3], Yang Gang[4], Taiping Zhang[4], Roberta Palorini[1,*], Ferdinando Chiaradonna[1,*]

Supplementary Figures

Figure S1. KRASmut MIA PaCa-2 cells show a higher glucose consumption than KRASwt BxPC-3.

Figure S2. Glc*N*Ac and mannose protect MIA PaCa-2 cells upon 2-DG treatment, without affecting the glycolysis.

Figure S3. Metabolic profile of PDAC cells.

Figure S4. Mannose protects KRASmut cells upon 2-DG treatment.

Figure S5. Mannose protects MIA PaCa-2 cells upon FR054 treatment.

Figure S6. Combined 48h-treatment of KRASmut MIA PaCa-2 cells with FR054 and the pan-RAS inhibitor BI-2852.

Figure S7. Combined treatment of KRASwt BxPC-3 cells with FR054 and the pan-RAS inhibitor BI-2852.

Figure S8. Combined treatment of KRASmut SU.86.86 and Capan-1 cells with FR054 and the pan-RAS inhibitor BI-2852.

Figure S9. Upon treatment with FR054 EGFR accumulates at ER and Golgi apparatus.

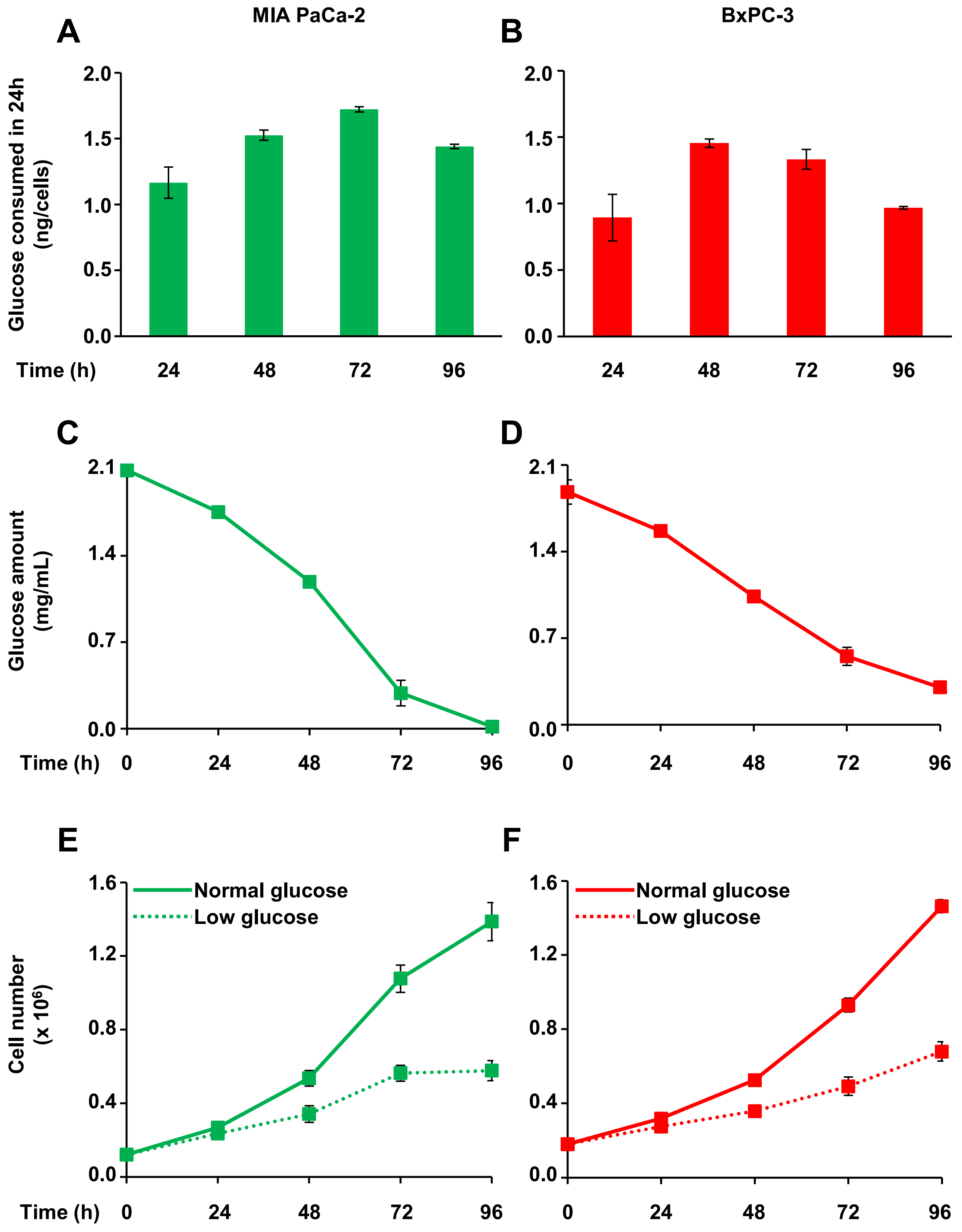

Figure S1. KRASmut MIA PaCa-2 cells show a higher glucose consumption than KRASwt BxPC-3. A-D) The 24h-glucose consumption (A-B) as well as the glucose depletion (C-D) along a time course of 96 hours was calculated measuring daily the amount of glucose in cell media of MIA PaCa-2 (A, C) and BxPC-3 (B, D). E-F) MIA PaCa-2 (E) and BxPC-3 (F) cells were cultured in normal (11 mM) and low (1mM) glucose conditions and counted at the indicated time points using Trypan Blue Stain. All data represent the mean ± SEM of at least three independent experiments.

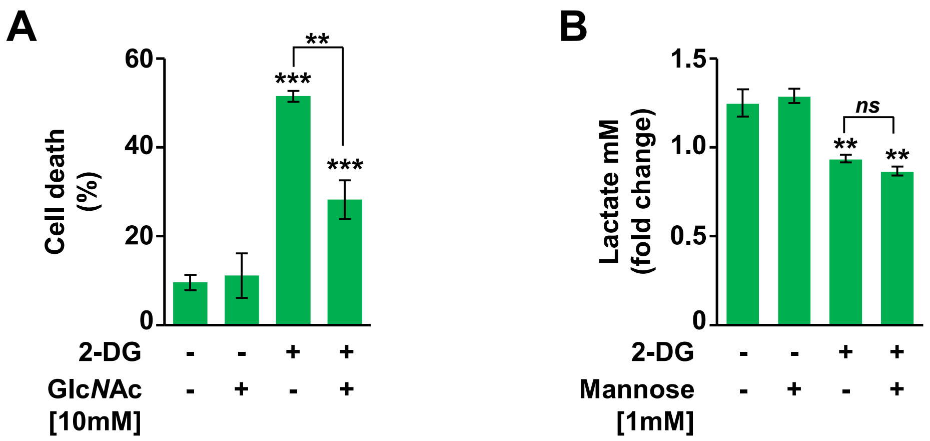

Figure S2. Glc*N*Ac and mannose protect MIA PaCa-2 cells upon 2-DG treatment, without affecting the glycolysis. A) Cell death of MIA PaCa-2 cells was evaluated through Trypan Blue assay upon 72h-treatment with 2.5 mM 2-DG -/+ 10 mM Glc*N*Ac. B) The lactate was measured in the media of MIA PaCa-2 cells upon 24h-treatment with 2.5 mM 2-DG -/+ 1 mM mannose. Data represent the mean ± SEM of three independent experiments. ** $P<0.01$, *** $P<0.001$, *ns* not significant (One-way ANOVA); untreated *vs.* treated where not specifically indicated.

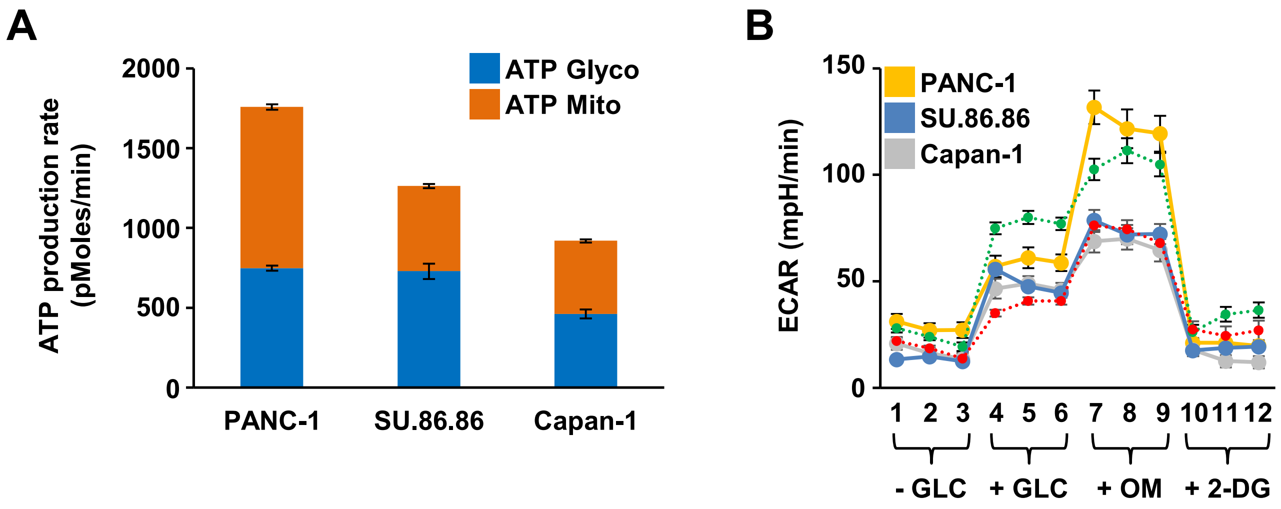

Figure S3. Metabolic profile of PDAC cells. A) The production rate of the ATP due to the glycolysis (ATP Glyco) and the mitochondrial respiration (ATP Mito) was evaluated in PANC-1, SU.86.86 and Capan-1 cells by performing the specific assay with the Seahorse XFe96 Analyzer. B) The ECAR of PANC-1, SU.86.86 and Capan-1 cells throughout a glycotest assay was measured using the Seahorse XF24 Analyzer. For an easier comparison, the profiles of BxPC-3 and MIA PaCa-2 already present in Figure 1A are shown here as dotted lines. The data represent the mean ± SEM.

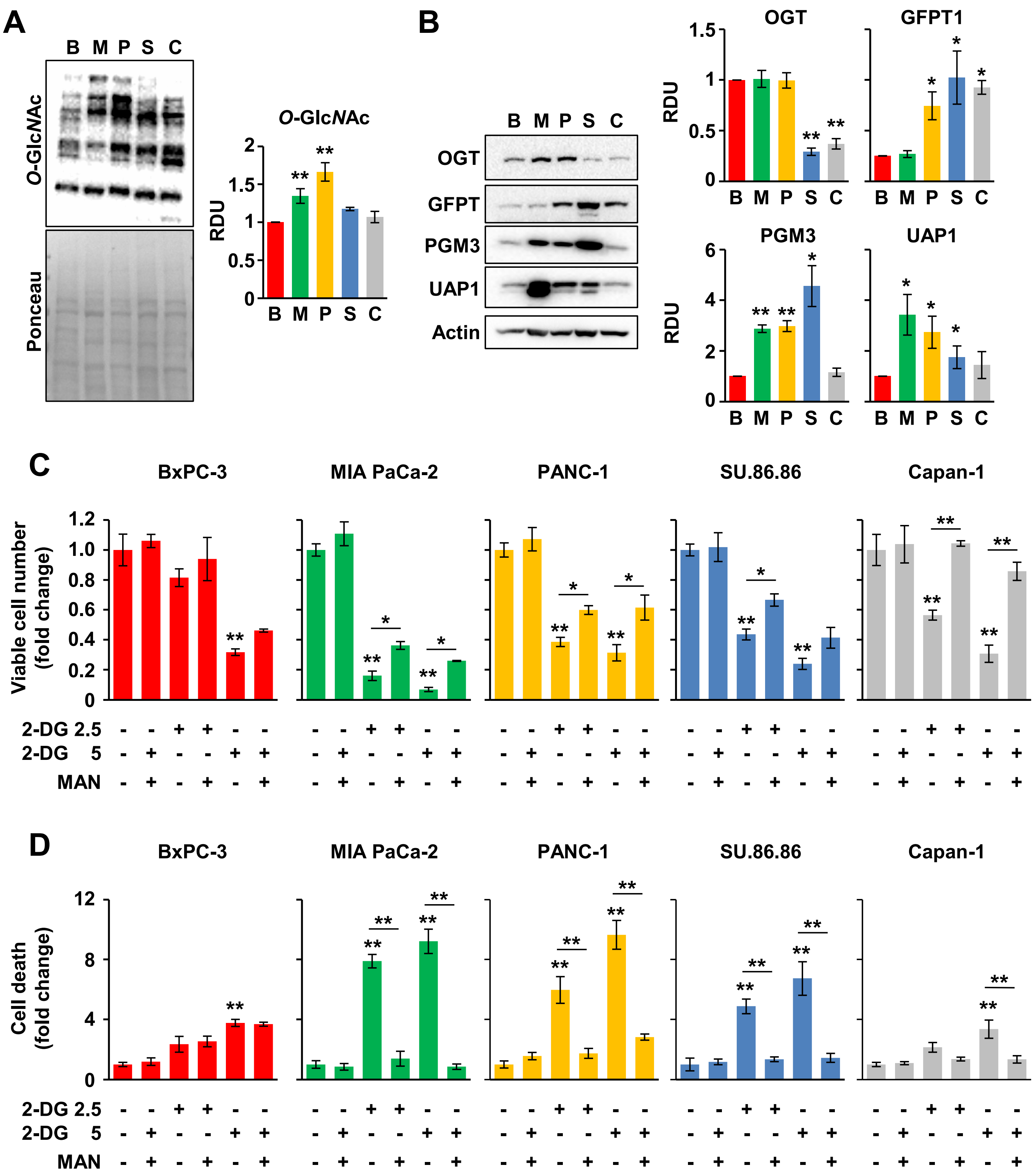

Figure S4. Mannose protects KRASmut cells upon 2-DG treatment. A-B) Protein *O*-Glc*N*Acylation levels (A) as well as the expression of the HBP proteins (B) were analyzed through Western blot in BxPC-3 [B], MIA PaCa-2 [M], PANC-1 [P], SU.86.86 [S] and Capan-1 [C] cells. As loading control the Ponceau staining (A) and the Actin expression (B) were used, respectively. On the left representative images are shown, while the band intensity quantification of at least two independent blots is displayed in the histograms on the right. C-D) Cell number (C) and cell death (D) of PDAC cells were evaluated through Trypan Blue assay upon 72h-treatment with 2.5 and 5 mM 2-DG -/+ 1 mM mannose. Data represent the mean ± SEM of three independent experiments. * P<0.05, ** P<0.01 (One-way ANOVA); untreated *vs.* treated where not specifically indicated.

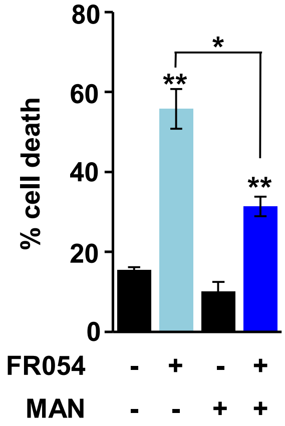

Figure S5. Mannose protects MIA PaCa-2 cells upon FR054 treatment. Cell death of MIA PaCa-2 treated with 0.5 mM FR054 and 1 mM mannose (MAN) was evaluated through Trypan Blue assay. Data represent the mean ± SEM of three independent experiments. * P<0.05, ** P<0.01 (One-way ANOVA); untreated *vs.* treated where not specifically indicated.

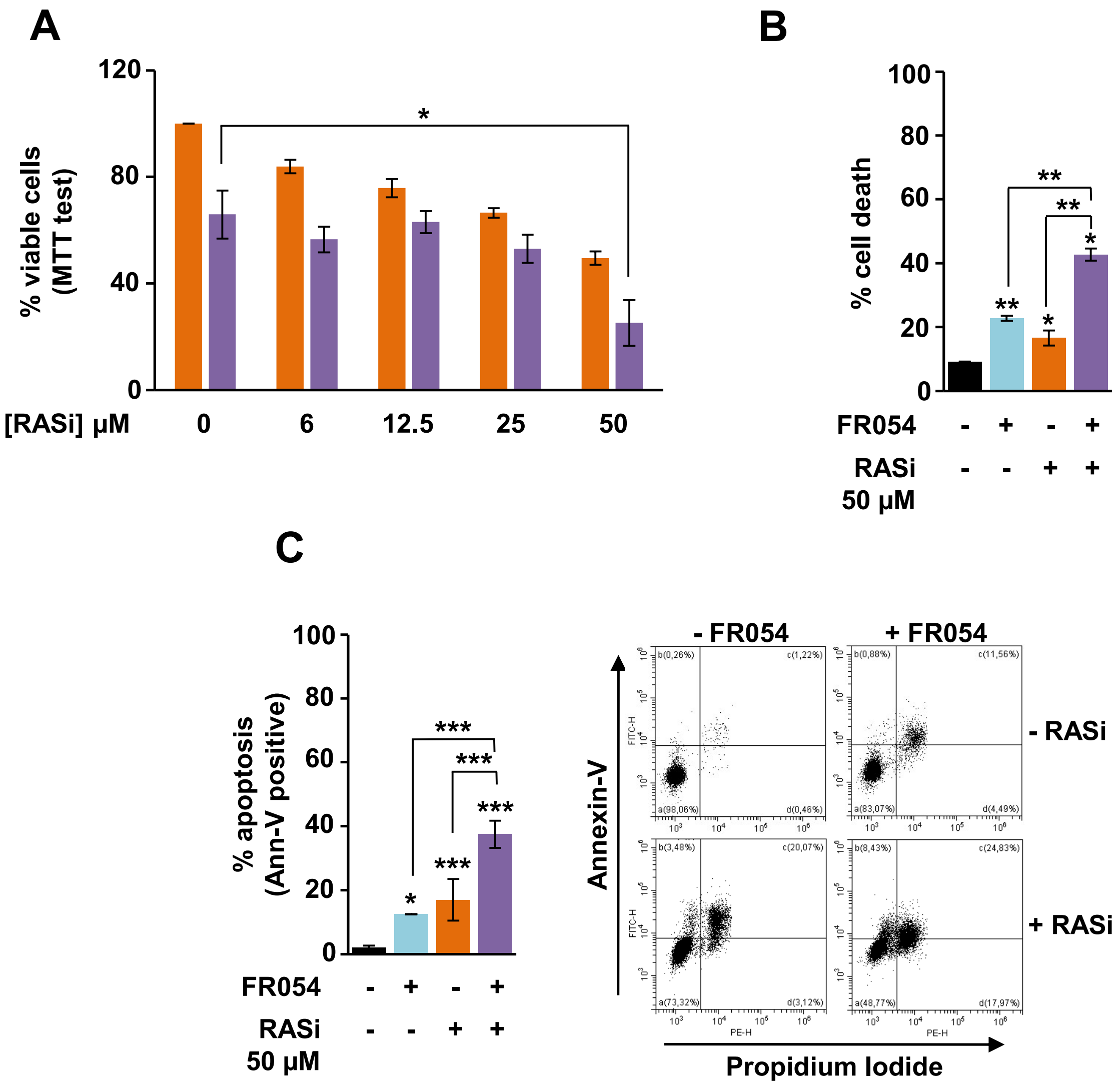

Figure S6. Combined 48h-treatment of KRASmut MIA PaCa-2 cells with FR054 and the pan-RAS inhibitor BI-2852. A) Cell viability of MIA-PaCa-2 treated for 48 hours with different concentrations of RAS inhibitor BI-2852 (RASi) and 350 µM FR054 was detected through MTT test. B) Cell death of MIA PaCa-2 treated for 48 hours with 50 µM BI-2852 and 350 µM FR054 was evaluated by viable count using Trypan Blue stain and C) by using flow cytometric analysis of Annexin V-FITC and propidium iodide staining. Representative profiles of the flow cytometric analysis are shown on the right of panel C. All data represent the mean ± SEM of at least three independent experiments. * P<0.05, ** P<0.01 and *** P<0.001 (One-way ANOVA), untreated *vs.* treated where not specifically indicated.

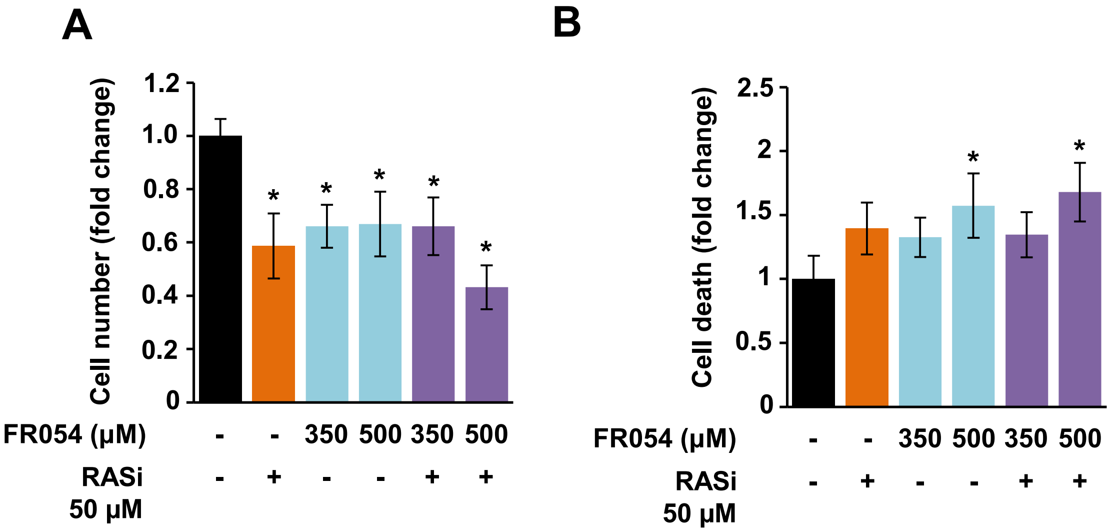

Figure S7. Combined treatment of KRASwt BxPC-3 cells with FR054 and the pan-RAS inhibitor BI-2852. A-B) Cell viability (A) and cell death (B) of BxPC-3 treated for 72 hours with the RAS inhibitor BI-2852 (RASi, 50 μM) and FR054 (350 μM or 500 μM) were evaluated by viable count using Trypan Blue stain. All data represent the mean ± SEM of two independent experiments with at least 3 replicates each. * $P<0.05$ (One-way ANOVA), untreated *vs.* treated where not specifically indicated.

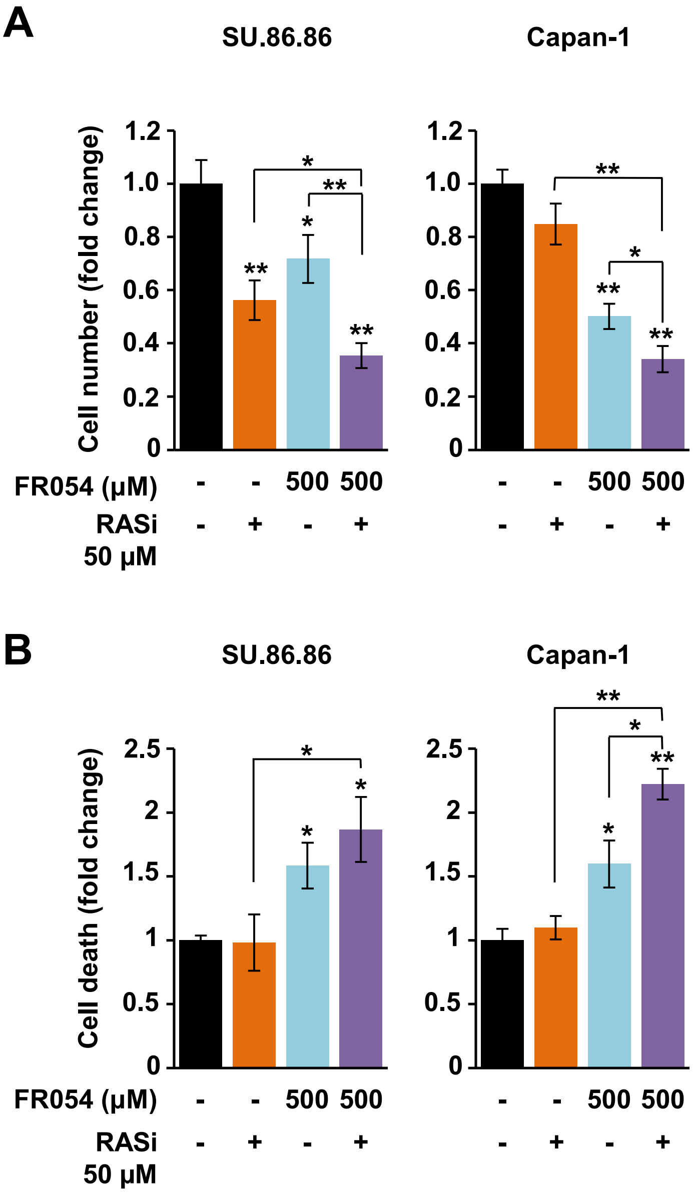

Figure S8. Combined treatment of KRASmut SU.86.86 and Capan-1 cells with FR054 and the pan-RAS inhibitor BI-2852. A-B) Cell viability (A) and cell death (B) of SU.86.86 and Capan-1 cells treated for 72 hours with the RAS inhibitor BI-2852 (RASi, 50 μM) and FR054 (500 μM) were evaluated by viable count using Trypan Blue stain. All data represent the mean ± SEM of two independent experiments with at least 3 replicates each. * $P<0.05$, ** $P<0.01$ (One-way ANOVA), untreated *vs.* treated where not specifically indicated.

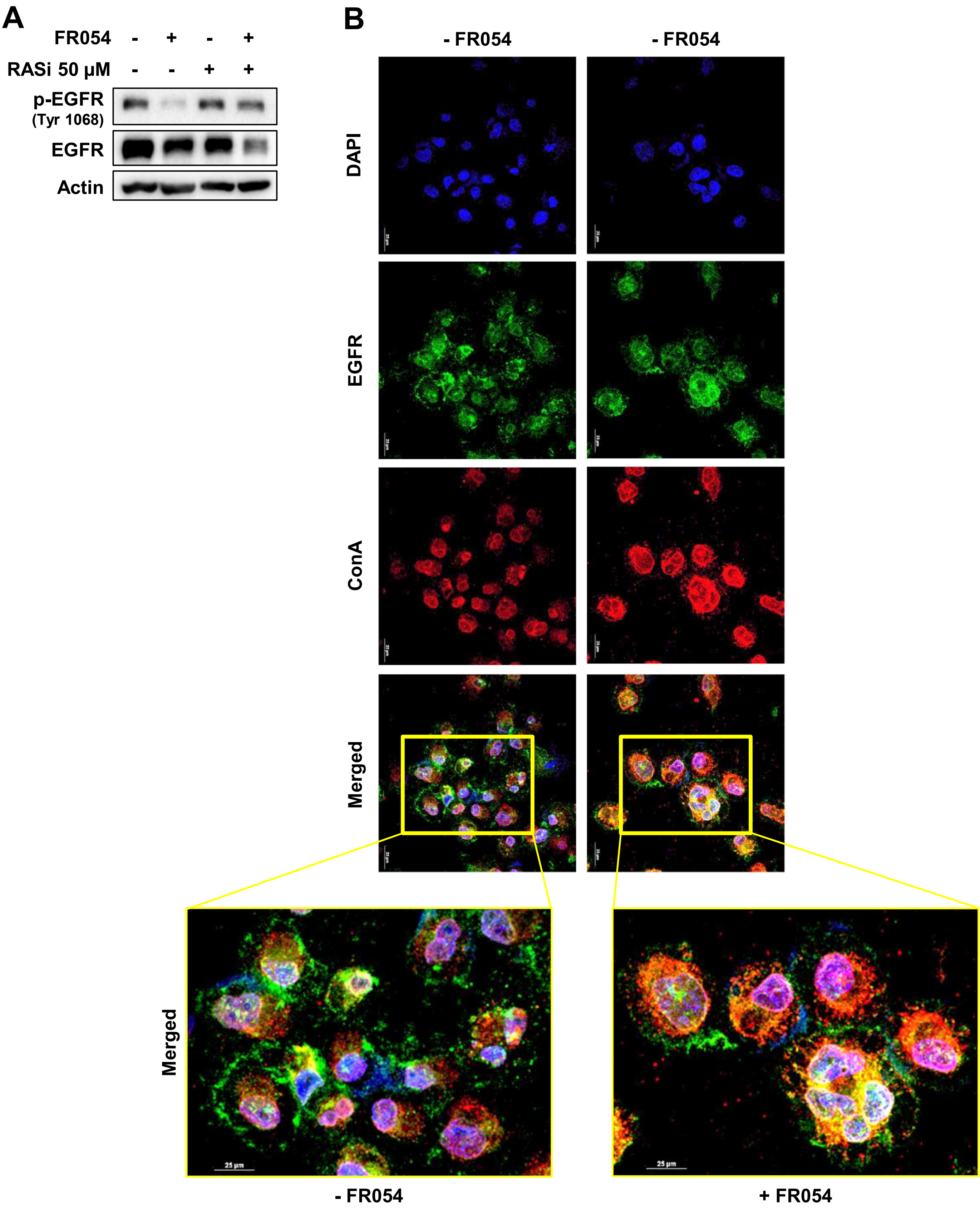

Figure S9. Upon treatment with FR054 EGFR accumulates at ER and Golgi apparatus. A) EGFR expression was analyzed through Western blot in MIA PaCa-2 cells treated with 50 μM BI-2852 and 350 μM FR054 for 48 hours. Actin was used for signal normalization. B) Confocal microscopy of total EGFR (green), Concanavalin-A (red) and DAPI (blue) staining in MIA PaCa-2 cells upon 24h-treatment with 350 μM FR054 (60X magnification, 25 μm scale).
